# Late Gadolinium Enhancement Variation in Asymptomatic Individuals: Comparison with Dilated Cardiomyopathy

**DOI:** 10.3390/jcdd12080312

**Published:** 2025-08-18

**Authors:** Seoyeon Park, Soo Jin Cho, Sung Mok Kim, Moon Young Kim, Yeon Hyeon Choe

**Affiliations:** 1 School of Medicine, Sungkyunkwan University, 2066, Seobu-ro, Jangan-gu, Suwon-si 16419, Republic of Korea; newhallen@naver.com; 2 Health Promotion Center, Samsung Medical Center, 81, Irwon-ro, Gangnam-gu, Seoul 06351, Republic of Korea; soojin77.cho@samsung.com; 3 Department of Radiology, Samsung Medical Center, 81, Irwon-ro, Gangnam-gu, Seoul 06351, Republic of Korea; 4 Cardiovascular Imaging Center, Heart Vascular and Stroke Institute, Samsung Medical Center, 81, Irwon-ro, Gangnam-gu, Seoul 06351, Republic of Korea; 5 Department of Radiology, Seoul Metropolitan Government Seoul National University, Boramae Medical Center, 20, Boramae-ro 5-gil, Dongjak-gu, Seoul 06351, Republic of Korea

**Keywords:** heart, cardiac magnetic resonance imaging, late gadolinium enhancement, dilated cardiomyopathy, asymptomatic

## Abstract

Late gadolinium enhancements (LGEs) appear in asymptomatic individuals as septal stripes, which mimic abnormal LGEs in patients with dilated cardiomyopathy (DCM). We aimed to evaluate the frequency and extent of LGE variation in asymptomatic individuals and to compare it with those of DCM group. This retrospective study included asymptomatic and DCM groups who underwent CMR imaging. LGE was defined as a myocardial signal intensity higher than five standard-deviations of normal myocardium. LGE was evaluated in right ventricular insertion points (RVIPs) and mid-interventricular septum. A total of 273 asymptomatic individuals (age, 54.3 ± 5.8 years, 209 males) and 100 patients with DCM (age, 55.3 ± 4.9 years, 73 males) were included. LGE was observed in 99.3% of asymptomatic and 100% of DCM groups. The average number of myocardial segments with LGE was distinguishable between asymptomatic and DCM groups (5.5 ± 1.7 vs. 7.6 ± 2.2; *p* < 0.001). The thickness of LGE differed between two groups in mid-septum (4.5 ± 1.3 mm vs. 5.7 ± 1.8 mm; *p* < 0.001), upper RVIP (6.1 ± 1.9 mm vs. 8.7 ± 2.7 mm; *p* < 0.001), and lower RVIP (6.4 ± 2.3 mm vs. 8.6 ± 2.8 mm; *p* < 0.001). Considerable overlap was observed in LGE between asymptomatic and DCM groups despite different LGE characteristics between them. LGEs within normal range should not be interpreted as abnormal findings in the evaluation of myocardial diseases including DCM.

## 1. Introduction

Late gadolinium enhancement (LGE) is a significant non-invasive cardiac magnetic resonance (CMR) imaging technique for detecting myocardial fibrosis, which is an important prognostic biomarker of cardiomyopathy. LGE is associated with various non-ischemic cardiomyopathies, especially dilated cardiomyopathy (DCM) [1,2].

In patients with DCM, the prevalence of LGE varies among reports with large patient populations ranging 39–82% [3,4,5,6,7,8,9]. The prognosis is known to be worse with higher amounts of LGE in patients with DCM. According to Behera et al, percentage of LGE over the myocardium of >16% in patients with DCM was associated with a worse prognosis than in patients with a lower LGE [10]. The frequency of LGE in DCM was highest in the mid-septum (69–93%) among left ventricular segments, followed by right ventricular insertion points (RVIPs, 33–60%) [11]. LGE in DCM appears as a striated pattern, particularly in the mid-septum, in 90% of patients [11].

T1 mapping and extracellular volume fraction (ECVF) have been found to exhibit differences in T1 relaxation time and ECVF values between the interventricular septum and other segments of the left ventricle in normal or asymptomatic individuals [12,13]. Interestingly, LGE is also found at the RVIP and middle interventricular septum in asymptomatic individuals. These variations in LGE in normal or asymptomatic individuals may mimic the pathological LGEs in patients with non-ischemic cardiomyopathy or myocarditis. According to recent studies, LGEs are detected in up to 38% of healthy athletes, especially in the RVIP [14,15,16,17]. However, there have been no reports on the prevalence and extent of LGE in asymptomatic individuals regardless of occupation that requires hard training. In this study, we aimed to evaluate variations in the myocardial LGE patterns in asymptomatic individuals and compare those with the LGE characteristics of patients with DCM to differentiate between the normal variation and pathological LGE.

## 2. Materials and Methods

### 2.1. Population

This retrospective study protocol was approved by the Institutional Review Board (IRB) of Samsung Medical Center. A waiver for informed consent was granted by the IRB considering the retrospective nature of the study. All methods followed the authorized guidelines and regulations.

**Figure 1:**
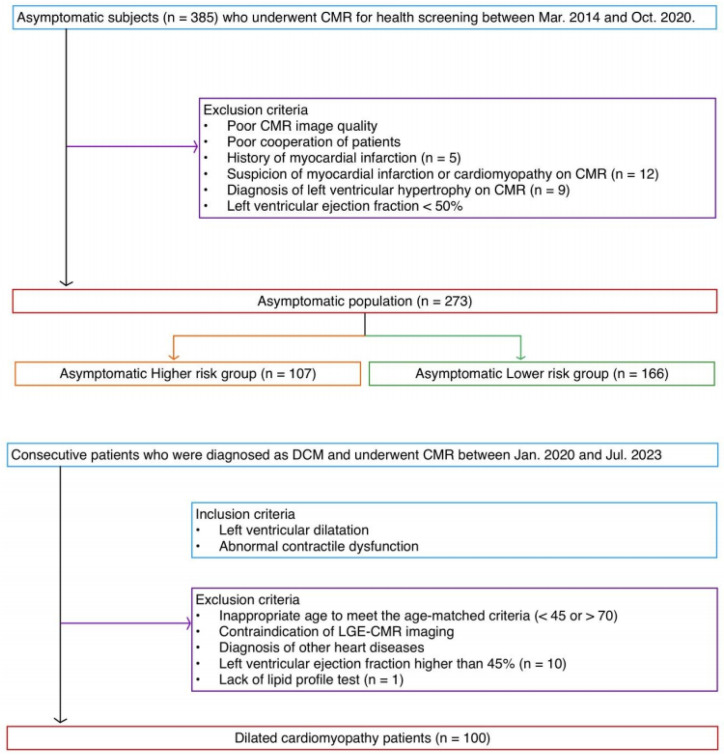
Flow chart of study population. The population of asymptomatic individuals was divided into two groups according to an ASCVD risk score of 7.5. Abbreviations: ASCVD, atherosclerotic cardiovascular disease; CMR, cardiac magnetic resonance; DCM, dilated cardiomyopathy; LGE, late gadolinium enhancement.

Data from asymptomatic individuals who underwent cardiac magnetic resonance (CMR) imaging for health screening were collected from the Samsung Medical Center Health Promotion Center between March 2014 and October 2020. The exclusion criteria were as follows: (1) poor image quality; (2) lack of patient cooperation; (3) history of myocardial infarction (*n* = 5); (4) suspicion of myocardial infarction or cardiomyopathy (*n* = 12); (5) left ventricular hypertrophy defined as wall thickness > 13 mm in at least one segment or an indexed end-diastolic mass > 91 g/m^2^ in males and > 77 g/m^2^ in females (*n* = 9); and, (6) left ventricular ejection fraction < 50%. After assessing participants against these criteria, a cohort of 273 asymptomatic individuals was included in this retrospective study (Figure 1). There is an overlap (*n* = 233) in the study population with a prior study [13]; however, the prior article reported T1 mapping and extracellular volume fraction, not LGE. As the prior study showed higher native T1 values and ECV in septal segments than those in lateral segments, in the current study, we evaluated the difference in LGE patterns in myocardial segments. Moreover, patients with DCM were included in the current study to compare the characteristics of LGE with the asymptomatic individuals.

The data of consecutive patients who had been diagnosed with DCM and underwent CMR were collected from the Samsung Medical Center, Department of Radiology between January 2020 and July 2023. Patients met the diagnostic criteria based on reduced left ventricular ejection fraction and increased left ventricular end-diastolic volume according to the World Health Organization/International Society and Federation of Cardiology [18]. Patients were excluded from the study owing to one or more following criteria: (1) contraindication to LGE-CMR imaging; (2) diagnosis of other heart diseases such as hypertrophic cardiomyopathy, infiltrative heart disease, arrhythmogenic right ventricular cardiomyopathy, acute myocarditis, or valvular heart disease; (3) left ventricular ejection fraction (LVEF) > 45% (*n* = 10); and, (4) lack of lipid profile test (*n* = 1). Ultimately, a total of 100 patients with DCM were included in this study (Figure 1).

### 2.2. CMR Technique

All study population underwent CMR using a 1.5 T MR system (MAGNETOM Avanto, Syngo MR D13, Siemens Healthineers, Erlangen, Germany) with a 32-channel phased-array receiver coil. Contiguous short-axial cine MR images (slice thickness, 6 mm; gap, 4 mm; image matrix, 272 × 192) covering the entire ventricles were obtained using the balanced steady-state free precession technique for ventricular function analysis. Standard delayed gadolinium enhancement imaging was performed 15–20 min after gadolinium contrast material (0.15 mmol/kg of gadobutrol) (Gadovist; Bayer Healthcare), using the phase-sensitive inversion recovery technique. Inversion delay times were typically 280–360 ms. 10–20 continuous short-axis slices of 6-mm thickness (image matrix, 256 × 190) at 4-mm intervals including 4-chamber, 3-chamber, and 2-chamber view images were obtained for LGE imaging.

### 2.3. Image Analysis

**Figure 2:**
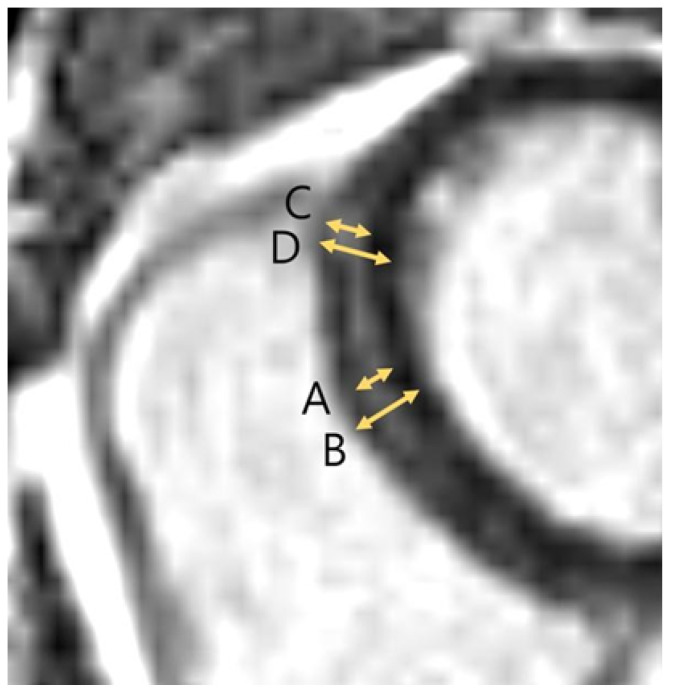
Measurement of LGE thickness and percent LGE thickness in a 52-year-old asymptomatic male. A. Thickness of late gadolinium enhancement in mid-septum. B. Full myocardial thickness in mid-septum. C. of late gadolinium enhancement in upper RVIP. D. Full myocardial thickness in upper RVIP. In the Thickness mid-septum, percent LGE thickness was measured as A/B. In the upper RVIP, percent LGE thickness was measured as C/D. Abbreviations: RVIP, right ventricular insertion point.

Image analysis was performed according to the consensus statement by the Society for Cardiovascular Magnetic Resonance (SCMR) [19]. Left ventricle function was assessed by an observer with 15 years of experience. The global LV function parameters, such as (1) left ventricular ejection fraction (LVEF), (2) left ventricular end-systolic volume index (LVESVi), (3) left ventricular end-diastolic volume index (LVEDVi), (4) left ventricular mass index (LVMi), and (5) right ventricular ejection fraction (RVEF) were calculated using dedicated software (Argus workstation, version 4.01) (Syngo.via, Siemens Healthineers, Erlangen, Germany). The LGE was defined as myocardial signal intensity higher than 5-SD of normal myocardium and enhancement assumed as artifacts, vessels, or muscles were excluded. LGE was measured by 2 observers in consensus on a cardiac MR-dedicated workstation (CVi42, release 5.16.0; Circle Cardiovascular Imaging Inc., Calgary, AB, Canada) and a picture-archiving communications workstation (Centricity, General Electric Healthcare, Barrington, IL, USA). Any discrepancies were resolved by a third observer with 21 years of expertise. LGE was measured in 16 left ventricular segments (except the apex, i.e., segment 17), classified by American Heart Association (AHA). The extent of LGE was determined by the maximal thickness and % LGE thickness compared with full myocardial thickness (Figure 2). The clinical information was blindfolded during the image analysis process.

### 2.4. Clinical Information

Clinical information of the individuals was derived from their medical records and laboratory findings on the day of CMR acquisition. The Atherosclerotic Cardiovascular Disease (ASCVD) 2013 risk calculator was used to calculate risk scores for the following variables: (1) age; (2) sex; (3) diabetes; (4) smoking status; (5) total cholesterol; (6) high-density lipoprotein cholesterol; (7) systolic blood pressure; and (8) hypertension treatment. The stratification of 10-year ASCVD risk scores was used to examine LGE variations in asymptomatic participants, with a standard score of 7.5. Other variables such as body mass index (BMI), alcohol consumption, electrocardiography (ECG), and N-terminal pro-B-type natriuretic peptide (NT-proBNP) level were included in the study.

### 2.5. Statistical Analysis

**Figure 3:**
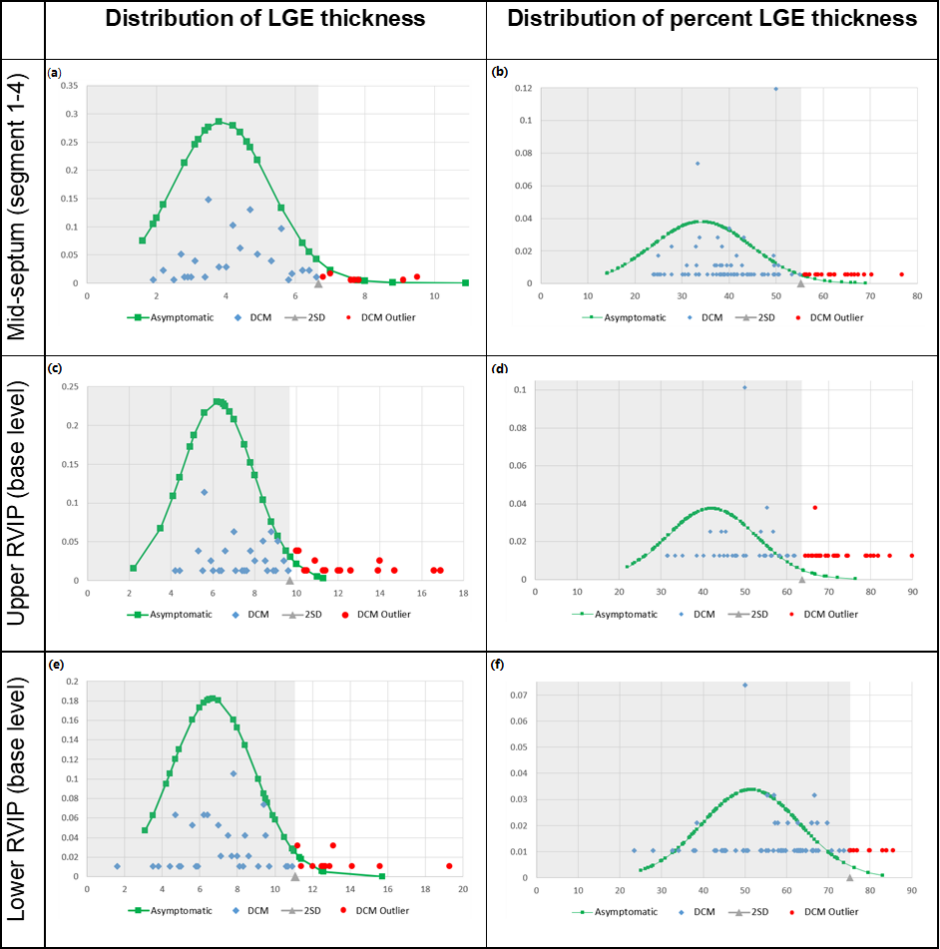
Distribution of LGE thickness (**a**,**c**,**e**) and percent LGE thickness (**b**,**d**,**f**) in asymptomatic individuals and patients with dilated cardiomyopathy according to LGE location. LGE thickness and % LGE thickness data of the asymptomatic individuals were plotted (square). The normal range is highlighted in gray on the graph, with the upper limit of the normal range indicated as the mean + 2SD of the asymptomatic individuals (triangle). The LGE thickness and % LGE thickness data of the patients with DCM were also plotted. The data of DCM group within the normal range (diamond) and above mean + 2SD (circle) were each displayed. Data are presented in the graph according to LGE location, where LGE was the most common (base level for RVIPs and segments 1–4 for the mid-septum). Abbreviations: SD, standard deviation.

All analyses were conducted using SPSS 26 (IBM, Chicago, IL, USA) and Rex software (version 3.0.3, RexSoft Inc., Seoul, Republic of Korea). Categorical variables are presented as percentages, whereas continuous variables are presented as means with standard deviations or within a range. Independent two-tailed *t*-tests were used to examine the differences between variables. Intraclass correlation coefficient (ICC) and its confidence interval (CI) were calculated between the observers to maintain the reliability of LGE measurement [20]. The normal range of LGE thickness and % thickness was defined as the (0, mean + 2SD) range in asymptomatic individuals (Figure 3). The overlap percentage between the asymptomatic and DCM groups was calculated using the normal range, which was set at the 95.4 percentile for asymptomatic individuals. Receiver operating characteristic (ROC) curves were drawn for LGE variation according to its location and extent (Figure S1). The cut-off values for pathological LGE were determined according to the Youden indexes of the ROC curves. Statistical significance was set at *p* = 0.05 (2 SD).

## 3. Results

### 3.1. Demographics

A total of 273 asymptomatic individuals (209 males and 64 females) and 100 patients (73 males and 27 females) with DCM were included in the study (Figure 1). The asymptomatic and DCM groups had similar age (54.3 ± 5.8 vs. 55.3 ± 4.9; *p* = 0.107) and sex (76.6% vs. 73%; *p* = 0.216), but they differed in BMI (24.5 ± 2.8 kg/m^2^ vs. 23.1 ± 3.8 kg/m^2^; *p* = 0.001) (Table 1). Smoking and hypertension had similar frequencies, whereas diabetes, hypercholesterolemia, and alcohol consumption differed. No significant difference was observed in the ASCVD risk score (7.6 ± 6.2 for asymptomatic vs. 8.4 ± 6.5 for DCM; *p* = 0.292). All cardiac function measurements were worse in DCM group than asymptomatic group. Differences were observed in LVEF (66.0 ± 5.5% vs. 26.5 ± 9.6%; *p* < 0.001), LVESVi (68.5 ± 10.0 mL/m^2^ vs. 167.0 ± 117.7 mL/m^2^; *p* < 0.001), LVEDVi (23.5 ± 5.9 mL/m^2^ vs. 116.8 ± 43.8 mL/m^2^; *p* < 0.001), LVMi (58.0 ± 10.9 g/m^2^ vs. 104.4 ± 100.3 g/m^2^; *p* < 0.001), RVEF (58.9 ± 6.3% vs. 41.7 ± 15.0%; *p* < 0.001), and NT-proBNP (27.2 ± 24.5 mg/dL vs. 2578.8 ± 3804.8 mg/dL; *p* < 0.001). LGE was observed in 99.3% of the asymptomatic individuals and in 100% of patients with DCM.

### 3.2. LGE Characteristics

Significant variations were observed in the LGE parameters between asymptomatic and DCM groups (Table 2). The average number of LGE segments was 5.5 ± 1.7 in asymptomatic individuals and 7.6 ± 2.2 in patients with DCM (*p* < 0.001). The maximal thickness and % thickness of LGE were significantly different between asymptomatic and DCM groups. In upper RVIP, maximal thickness was 6.1 ± 1.9 mm vs. 8.7 ± 2.7 mm (*p* < 0.001), while % thickness was 42.5 ± 10.6% vs. 57.8 ± 15.8% (*p* < 0.001). In mid-septum, maximal thickness was 4.5 ± 1.3 mm vs. 5.7 ± 1.8 mm (*p* < 0.001), while % thickness was 33.3 ± 9.8% vs. 42.9 ± 11.1% (*p* < 0.001). In lower RVIP, maximal thickness was 6.4 ± 2.3 mm vs. 8.6 ± 2.8 mm (*p* < 0.001), while % thickness was 48.2 ± 12.6% vs. 57.5 ± 12.6% (*p* < 0.001).

### 3.3. Overlap Percentage of LGE Extent

Patients with DCM overlapped with asymptomatic individuals in terms of the degree of myocardial LGE involvement according to the maximal thickness and % thickness (Table 3, Figure 3). The data of DCM group within the (0, mean + 2SD) range of the asymptomatic group was defined as an overlap. The overlap percentage of between two groups were 79.8% in LGE thickness and 81.1% in LGE % thickness, overall. The overlapping in LGE thickness and LGE % thickness were present in mid-septum in 80.7% and 82.1%, respectively; upper RVIP in 75.3% and 63.4%, respectively; and lower RVIP in 80.7% and 88.4%, respectively.

### 3.4. Cut-Off Value for Pathological LGE

A total of 6 LGE variations were analyzed according to its location (mid-septum, upper RVIP, and lower RVIP) and its extent (maximal thickness and % thickness). ROC curves were drawn and cut-off values for each curve were calculated, using their Youden indexes (Figure S1). The optimal cut-off values for pathological LGE thickness and % thickness were estimated to be 5.5 mm and 46.8% for mid-septum; 8.5 mm and 56.1% for upper RVIP; and 9.5 mm and 66.0% for lower RVIP.

### 3.5. ASCVD Risk Score

According to the 10-year ASCVD risk score stratification, LGE differences among asymptomatic participants were statistically significant (Table S1). The average number of LGE segments between higher-risk (score≥7.5) and lower-risk groups were significantly different (5.8 ± 1.8 vs. 5.3 ± 1.5; *p* = 0.026). LGE was significantly thicker in higher-risk than lower-risk group in mid-septum (4.8 ± 1.2 mm vs. 4.4 ± 1.3 mm; *p* = 0.003) and lower RVIP (7.1 ± 2.4 mm vs. 5.9 ± 2.1 mm; *p* < 0.001) but not in upper RVIP (6.3 ± 1.7 mm vs. 6.0 ± 1.9 mm; *p* = 0.144). Percentage thickness of LGE were greater in higher-risk than lower-risk group in lower RVIP (50.2 ± 12.5% vs. 47.0 ± 12.5%; *p* = 0.004) while it was not distinguishable in upper RVIP (41.9 ± 10.2% vs. 43.0 ± 10.8%; *p* = 0.396) and mid-septum (33.0 ± 10.1% vs. 33.5 ± 9.5%; *p* = 0.553).

### 3.6. LGE Distribution

**Figure 4:**
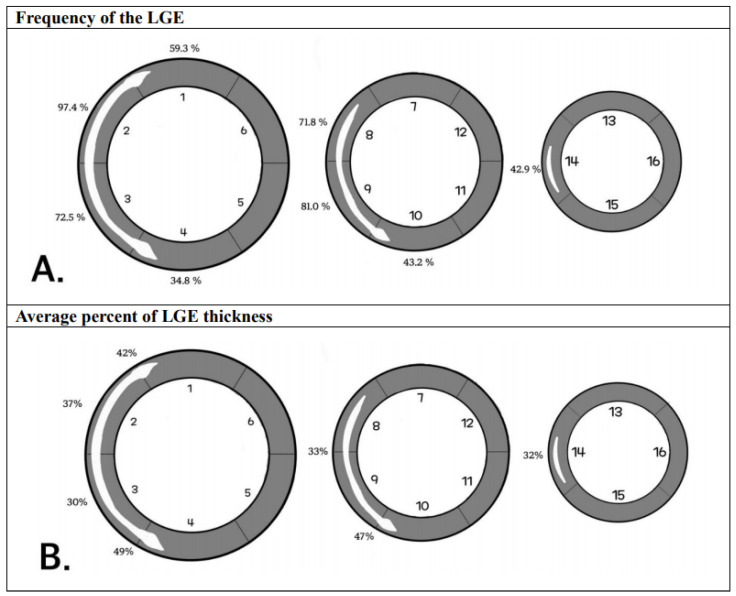
Typical LGE appearance, frequency of LGE, and average percent LGE thickness according to myocardial segments in the asymptomatic individuals. Typical appearance of LGE variation in the asymptomatic individuals is drawn. (**A**) The frequencies (%) of LGEs in AHA segments are presented. (**B**) Average percents of LGE thickness are presented in each segment. Abbreviations: AHA, American heart association.

The typical appearance of LGE in asymptomatic individuals showed that LGE was distributed mostly in the mid-septum and RVIPs (Figure 4). LGE was shown as stripes in the mid-septum while triangular patches were detected in the RVIPs. LGE was present in more than a one-quarter of the asymptomatic individuals in segments 1 (59.3%), 2 (97.4%), 3 (72.5%), 4 (34.8%), 8 (71.8%), 9 (81.0%), 10 (43.2%), and 14 (42.9%).

**Figure 5:**
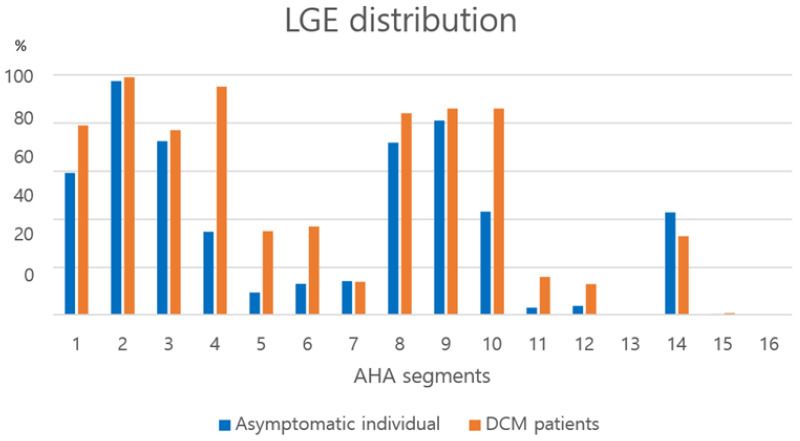
Frequencies of LGEs according to the AHA segments in the asymptomatic individuals and patients with dilated cardiomyopathy. LGE segments in asymptomatic individuals and patients with DCM were analyzed by location according to the 16 segments of AHA.

**Figure 6:**
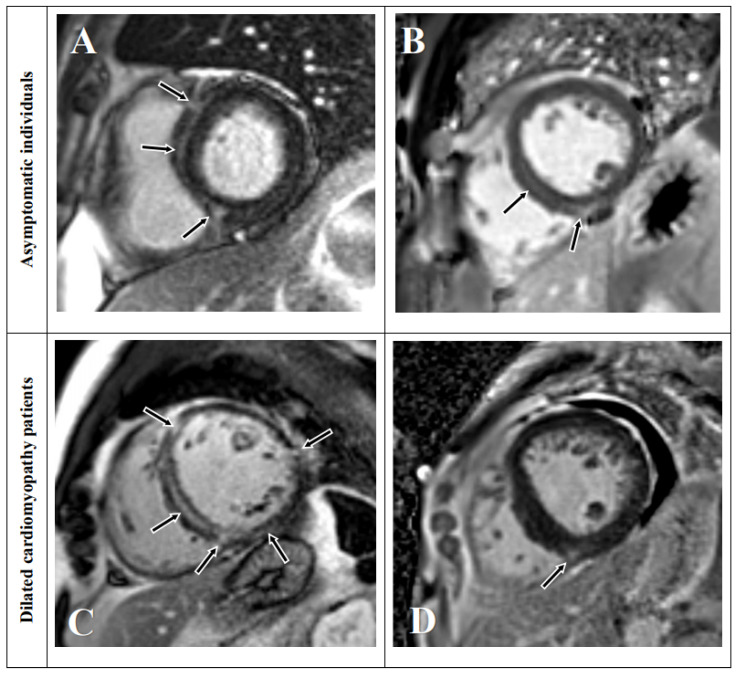
Late gadolinium enhancement patterns (arrows) in the asymptomatic individuals and patients with DCM. (**A**) A 54-year-old asymptomatic male individual with normal LVEF (61.3%) showed late gadolinium enhancement in the mid-septum and RVIPs. (**B**) A 54-year-old asymptomatic male individual with normal LVEF (66%) showed late gadolinium enhancement in the mid-septum and lower RVIP. (**C**) A 60-year-old dilated cardiomyopathy male patient with severely decreased LVEF (19%) showed late gadolinium enhancement in all segments. (**D**) A 63-year-old dilated cardiomyopathy female patient with slightly decreased LVEF (42.8%) showed late gadolinium enhancement in the lower RVIP. Abbreviations: LVEF, left ventricular ejection fraction.

LGE was frequently present in mostly mid-septum and RVIPs in asymptomatic individuals, whereas LGE in lateral (segments 5, 6, 11, and 12) and apex (segments 13, 15, and 16) were rare (Figure 5). LGE was distributed in nearly all locations except the apex (segments 13, 15, and 16) in patients with DCM. Although, LGE patterns of asymptomatic and DCM groups differed according to their cardiac function parameters such as LVEF (Figure 6). The observers showed high ICC based on a mean-rating, absolute-agreement, 2-way random-effects model in evaluating: 0.937 in asymptomatic individuals (95% CI; 0.856–0.975) and 0.965 in DCM patients (95% CI; 0.917–0.986) as for the measurement of LGE thickness and LGE % thickness.

## 4. Discussion

In the present study, we retrospectively reported the normal features of LGE patterns in asymptomatic individuals and compared them with those in patients with DCM. LGE was present in 99.3% of asymptomatic individuals and in all patients with DCM. LGEs in patients with DCM differed from those in asymptomatic individuals in terms of the average number, distribution, maximal thickness, and % thickness of LGE. However, LGE measurements in around 80% of the patients with DCM overlapped within the (0, mean + 2SD) range of those in the asymptomatic individuals. The cut-off values for pathological LGE were analyzed according to LGE thickness and percentage of LGE over normal myocardium (mid-septum, 5.5 mm and 46.8%; upper RVIP, 8.5 mm and 56.1%; lower RVIP, 9.5 mm and 66.0%). This suggests that LGE should be interpreted carefully in patients with DCM.

The RVIP was defined as the junction of the right ventricle meeting the anterior or posterior ventricular septum [21]. In consensus with other studies, the study found a higher frequency of LGE in the RVIP in both groups [11,21,22,23]; thus, LGE detected in the RVIP needs to be carefully evaluated. A recent study showed that LGE in RVIP is merely a gadolinium aggregation in the greatly expanded extracellular space between ventricles [22]. While LGE in RVIP is commonly observed in patients with pulmonary artery hypertension [24,25], several studies pointed out that LGE was frequently detected in the athletic population especially in RVIP region (11-38%) because repetitive hard training caused pulmonary artery pressure overload which eventually formed fibrosis in the RVIPs [14,15,16,17].

The presence of LGE located in the RVIP did not have a significant effect on cardiovascular outcomes compared with the absence of LGE in patients with suspected pulmonary hypertension [25]. The fact that LGE extent in RVIPs overlapped significantly between asymptomatic and DCM groups is in line with these results. In a recent study, LGE in the lower RVIP was found to be triangular shape, owing to the unique arrangement of aggregate cardiomyocytes [23], which was consistent with our study. However, the authors reported that the orientation of aggregate cardiomyocytes was present only in the lower RVIP and absent in the upper RVIP [23], while the present study revealed that the frequency of LGE in the lower and upper RVIPs at the base level (93.0% vs. 84.7%) and the middle level (89.5% vs. 14.2%) are quite different. Myocardial parametric mapping show that septal segments have higher T1 and ECV values than lateral segments [13]. The results of the present study suggest that the high frequency of LGE in the mid-septum and RVIPs may be associated with higher T1 and ECV values in these specific areas.

LGE is most commonly found in the mid-septum in a striped pattern in patients with DCM [11]. However, the prognostic value of septal LGE in myocarditis and DCM remains controversial. According to recent studies, septal LGE has prognostic value in non-ischemic cardiomyopathy (NICM) patients [1,26,27]. In patients with NICM, septal fibrosis is crucial for predicting the probability of arrhythmia and determining the need for implantable cardioverter defibrillator (ICD) therapy [26]. In contrast, Mulla et al. insisted that anteroseptal LGE does not have any relevance to unfavorable outcomes in patients with acute myocarditis [28]. The controversy regarding the prognostic significance of mid-septal LGE may be explained by the results of our study; most asymptomatic individuals had LGEs, primarily in the mid-septum. This indicates that the clinical significance of septal LGE in patients with NICM should be closely evaluated before planning preventive ICD therapies.

Several studies have insisted that septal vessels are misinterpreted as septal LGEs [29,30]. Nevertheless, the anterior septal perforator artery extends from the proximal left anterior descending artery to the upper two-thirds of the interventricular septum and does not reach the inferior septum. In this study, the asymptomatic individuals had mid-septal LGE commonly connected in AHA segments 2, 3, and 4, which resembled the typical linear septal fibrosis in patients with DCM. Additionally, whereas LGE in the present study was displayed continuously throughout multiple sections, the septal vessels were only recognized in a single section. The vessels and septal LGE occasionally appear discontinuous and separate on CMR images, and their signal intensity differ significantly.

High frequencies of LGEs in both asymptomatic and DCM groups in our study might be the result of careful observation of basal septal and middle inferoseptal LGEs, that were the foci of attention in this study.

This study has some limitations. First, the sample size was relatively small. Second, the baseline data (alcohol consumption, BMI, hypercholesterolemia, diabetes) between the asymptomatic and DCM groups were different.

## 5. Conclusions

In conclusion, this study found that LGEs were found in most asymptomatic individuals. However, their characteristics differed from those in patients with DCM. A considerable overlap was observed in septal or RVIP LGE between the asymptomatic and DCM groups. These findings suggest that RVIP and septal LGEs in patients with DCM should be interpreted carefully to avoid an overestimation of LGE extent in these patients.

## Figures and Tables

**Table 1:** Demographics of asymptomatic and DCM groups.

Characteristics	Asymptomatic Individuals (*n* = 273)	DCM Patients (*n* = 100)	*p*-Value
Age (Y)	54.3 ± 5.8	55.3 ± 4.9	0.107
Age range (Y)	[44, 82]	[47, 70]	
Male (%)	209 (76.6%)	73 (73%)	0.216
BMI (kg/m^2^)	24.5 ± 2.8	23.1 ± 3.8	0.001
Hypertension	103 (37.7%)	37 (37%)	0.948
Diabetes	57 (20.9%)	33 (33%)	0.008
Hypercholesterolemia	98 (35.9%)	22 (22%)	0.002
Smoking	152 (55.7%)	59 (59%)	0.567
Alcohol use	212 (77.7%)	46 (46%)	<0.001
Atrial fibrillation	0	14 (14%)	
LBBB	0	7 (7%)	
ASCVD risk score	7.6 ± 6.2	8.4 ± 6.5	0.292
Low risk (0–4.9)	112 (41.0%)	41 (41%)	
Borderline risk (5–7.4)	54 (19.8%)	14 (14%)	
Intermediate risk (7.5–20)	93 (34.1%)	38 (38%)	
High risk (>20)	14 (5.1%)	7 (7%)	
LVEF (%)	66.0 ± 5.5	26.5 ± 9.6	<0.001
LVESVi (mL/m^2^)	68.5 ± 10.0	167.0 ± 117.8	<0.001
LVEDVi (mL/m^2^)	23.5 ± 5.9	116.8 ± 43.8	<0.001
LVMi (g/m^2^)	58.0 ± 10.9	104.4 ± 100.3	<0.001
RVEF (%)	58.9 ± 6.3	41.7 ± 15.0	<0.001
NT-proBNP (mg/dL)	27.2 ± 24.5	2578.8 ± 3804.8	<0.001
LGE presence (%)	99.267	100	

**Table 2:** LGE characteristics in asymptomatic and DCM groups.

LGE Characteristics	Asymptomatic Individuals (*n* = 273)	DCM Patients (*n* = 100)	*p*-Value
Average number of LGE segments	5.5 ± 1.7	7.6 ± 2.2	<0.001
Maximal thickness of upper RVIP LGE stripes	6.1 ± 1.9 mm (*n* = 239)	8.7 ± 2.7 mm (*n* = 80)	<0.001
% thickness of upper RVIP basal LGE stripes	42.2 ± 10.6% (*n* = 237)	57.5 ± 13.0% (*n* = 79)	<0.001
% thickness of upper RVIP middle LGE stripes	44.3 ± 10.1% (*n* = 39)	59.4 ± 15.8% (*n* = 14)	0.005
% thickness of upper RVIP LGE stripes	42.5 ± 10.6%	57.8 ± 13.4%	<0.001
Maximal thickness of mid-septal LGE stripes	4.5 ± 1.3 mm (*n* = 270)	5.7 ± 1.8 mm (*n* = 100)	<0.001
% thickness of mid-septal segment 1, 2 stripes	37.0 ± 9.5% (*n* = 267)	44.3 ± 10.4% (*n* = 99)	<0.001
% thickness of mid-septal segment 3, 4 stripes	29.5 ± 9.3% (*n* = 197)	39.9 ± 9.9% (*n* = 77)	<0.001
% thickness of mid-septal segment 8, 9 stripes	32.9 ± 9.8% (*n* = 239)	44.2 ± 12.2% (*n* = 87)	<0.001
% thickness of mid-septal segment 14 stripes	32.2 ± 7.9% (*n* = 117)	42.6 ± 11.7% (*n* = 33)	<0.001
% thickness of mid-septal stripes	33.3 ± 9.8%	42.9 ± 11.1%	<0.001
Maximal thickness of lower RVIP LGE stripes	6.4 ± 2.3 mm (*n* = 266)	8.6 ± 2.8 mm (*n* = 99)	<0.001
% thickness of lower RVIP basal LGE stripes	49.4 ± 12.6% (*n* = 252)	58.5 ± 12.3% (*n* = 95)	<0.001
% thickness of lower RVIP middle LGE stripes	47.1 ± 12.5% (*n* = 248)	56.3 ± 13.0% (*n* = 86)	<0.001
% thickness of lower RVIP LGE stripes	48.2 ± 12.6%	57.5 ± 12.6%	<0.001

**Table 3:** Overlap of LGE extent between asymptomatic and DCM groups.

	LGE Thickness	% LGE Thickness
Mid-septum	80.7%	82.1%
Segment 1, 2 (n = 99)	94.9% (94/99)	100% (99/99)
Segment 3, 4 (n = 77)	85.7% (66/77)	72.7% (56/77)
Segment 8, 9 (n = 87)	74.7% (65/87)	78.2% (68/87)
Segment 14 (n = 33)	42.4% (14/33)	60.6% (20/33)
Upper RVIP	75.3%	63.4%
Upper RVIP base level (n = 79)	73.4% (58/79)	64.6% (51/79)
Upper RVIP middle level (n = 14)	85.7% (12/14)	57.1% (8/14)
Lower RVIP	80.7%	88.4%
Lower RVIP base level (n = 95)	84.2% (80/95)	89.5% (85/95)
Lower RVIP middle level (n = 86)	76.7% (66/86)	87.2% (75/86)
Overall	79.8%	81.1%

## Data Availability

The raw data supporting the conclusions of this article will be made available by the authors on request.

## Supplementary Materials

The following supporting information can be downloaded at: https://www.mdpi.com/article/10.3390/jcdd12080312/s1, Figure S1: ROC curve of LGE variation between the asymptomatic and DCM groups. Six ROC curves of LGE variation between the asymptomatic and DCM groups. Abbreviations: ROC, receiver operating characteristic; LGE, late gadolinium enhancement; DCM, dilated cardiomyopathy; RVIP, right ventricular insertion point; AUC, area under curve. Table S1: LGE characteristics in asymptomatic subjects, stratified by ASCVD risk score.

## Abbreviations

The following abbreviations are used in this manuscript:

AHA	American Heart Association
ASCVD	Atherosclerotic cardiovascular disease
CMR	Cardiac magnetic resonance
DCM	Dilated cardiomyopathy
LGE	Late gadolinium enhancement
LVEF	Left ventricular ejection fraction
RVIP	Right ventricular insertion point
