# Supplementary material for: Late Gadolinium Enhancement Variation in Asymptomatic Individuals: Comparison with Dilated Cardiomyopathy
Source: J Cardiovasc Dev Dis. 2025 Aug 18;12(8):312. doi: 10.3390/jcdd12080312 (PMC12386344; doi:10.3390/jcdd12080312)
Supplement: Supplementary file 1 [file jcdd-12-00312-s001.zip › jcdd-3794122-supplementary.pdf]

Supplementary Material

Figure S1. ROC curve of LGE variation between the asymptomatic and DCM groups

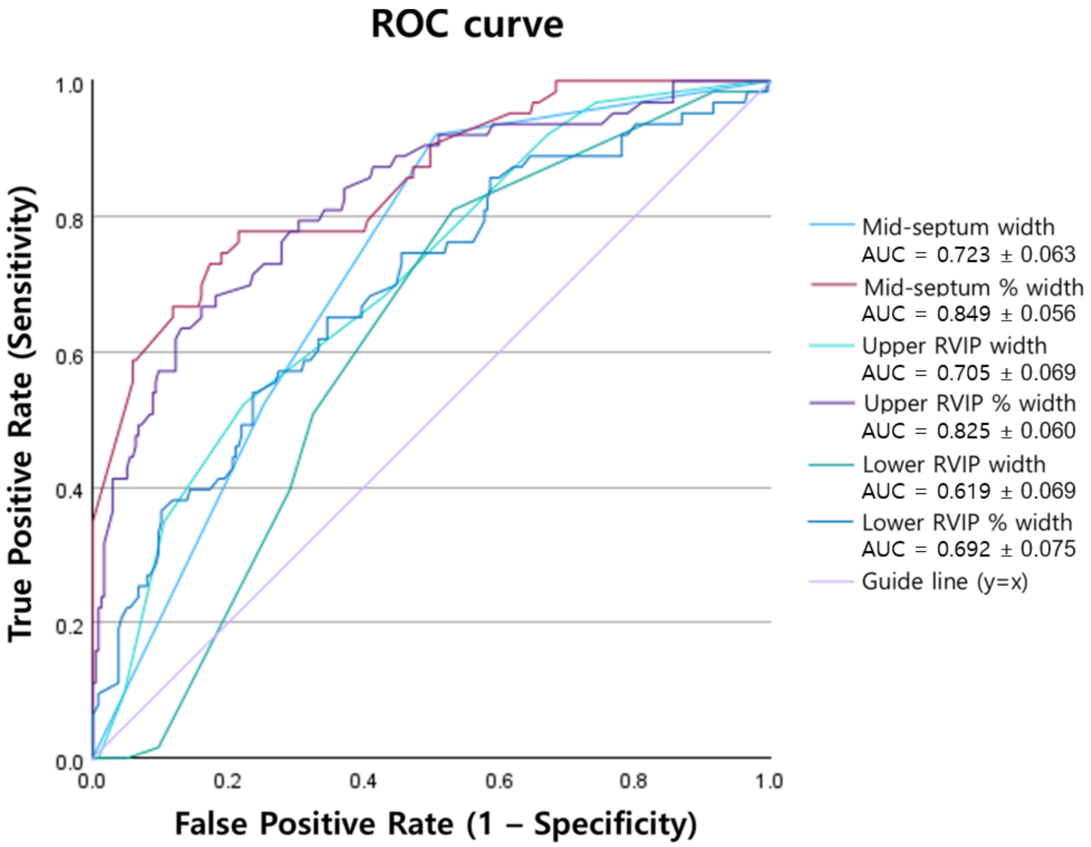

Six ROC curves of LGE variation between the asymptomatic and DCM groups.

Abbreviations: ROC, receiver operating characteristic; *LGE*, late gadolinium enhancement; *DCM*, dilated cardiomyopathy; *RVIP*, right ventricular insertion point; *AUC,* area under curve

Table S1. LGE characteristics in asymptomatic subjects, stratified by ASCVD risk score.

Characteristics	Asymptomatic, higher risk (n = 107)	Asymptomatic, lower risk (n = 166)	*p*-value
**Average number of LGE segments**	5.8 ± 1.8	5.3 ± 1.5	0.026
**Maximal thickness of upper RVIP LGE stripes**	6.3 ± 1.7 mm (n = 98)	6.0 ± 1.9 mm (n = 141)	0.144
**% thickness of upper RVIP Basal LGE stripes**	41.8 ± 10.6% (n = 96)	42.6 ± 10.7% (n = 141)	0.573
**% thickness of upper RVIP Mid LGE stripes**	42.5 ± 7.9% (n = 19)	45.9 ± 11.8% (n = 20)	0.299
**% thickness of upper RVIP LGE stripes**	41.9 ± 10.2%	43.0 ± 10.8%	0.396
**Maximal thickness of mid-septal LGE stripes**	4.8 ± 1.2 mm (n = 105)	4.4 ± 1.3 mm (n = 165)	0.003
**% thickness of mid-septal segment 1, 2 stripes**	37.1 ± 10.2% (n = 105)	36.8 ± 9.1% (n = 162)	0.842
**% thickness of mid-septal segment 3, 4 stripes**	28.1 ± 8.7% (n = 78)	30.4 ± 9.6% (n = 119)	0.089
**% thickness of mid-septal segment 8, 9 stripes**	33.5 ± 10.3% (n = 96)	32.5 ± 9.5% (n = 143)	0.441
**% thickness of mid-septal segment 14 stripes**	31.1 ± 7.3% (n = 44)	32.8 ± 8.2% (n = 73)	0.230
**% thickness of mid-septal stripes**	33.0 ± 10.1%	33.5 ± 9.5%	0.553
**Maximal thickness of lower RVIP LGE stripes**	7.1 ± 2.4 mm (n = 104)	5.9 ± 2.1 mm (n = 162)	< 0.001
**% thickness of lower RVIP Basal LGE stripes**	52.3 ± 11.6% (n = 98)	47.6 ± 12.8% (n = 154)	0.003
**% thickness of lower RVIP Mid LGE stripes**	48.2 ± 13.0% (n = 99)	46.3 ± 12.1% (n = 149)	0.255
**% thickness of lower RVIP LGE stripes**	50.2 ± 12.5%	47.0 ± 12.5%	0.004

Asymptomatic subjects were divided into two groups based on a 10-year ASCVD risk score of 7.5. The higher-risk and lower-risk groups did not show significant differences in the LGE segments or maximal thickness of the LGE stripes.

Values are presented as the mean ± SD.

Abbreviations: ASCVD, atherosclerotic cardiovascular disease; *LGE*, late gadolinium enhancement; *RVIP*, right ventricular insertion point; *SD*, standard deviation
